# Fiducial markers in adjuvant setting for a patient affected by endometrial cancer: a case report

**DOI:** 10.3389/fonc.2023.1174675

**Published:** 2023-08-25

**Authors:** Francesca Titone, Stefano Restaino, Eugenia Moretti, Gianluca Vullo, Alice Poli, Martina Arcieri, Chiara Paglietti, Fabrizio Tonetto, Giuseppe Parisi, Elisa Barbui, Marco Trovò, Giovanni Scambia, Lorenza Driul, Giuseppe Vizzielli

**Affiliations:** ^1^ Radiation Oncology Unit, Department of Oncology, “Santa Maria della Misericordia” University Hospital, Azienda Sanitaria Universitaria Friuli Centrale, Udine, Italy; ^2^ Clinic of Obstetrics and Gynecology, “Santa Maria della Misericordia” University Hospital, Azienda Sanitaria Universitaria Friuli Centrale, Udine, Italy; ^3^ Medical Physics Unit, Department of Oncology, “Santa Maria della Misericordia” University Hospital, Azienda Sanitaria Universitaria Friuli Centrale, Udine, Italy; ^4^ Department of Medicine, University of Udine, Udine, Italy; ^5^ Department of Biomedical, Dental, Morphological and Functional Imaging Science, University of Messina, Messina, Italy; ^6^ Dipartimento per le Scienze Della Salute Della Donna, del Bambino e di Sanità Pubblica, Unità Operativa Complessa (UOC) Ginecologia Oncologica, Fondazione Policlinico Universitario Agostino Gemelli Istituto di Ricovero e Cura a carattere Scientifico (IRCCS), Rome, Italy

**Keywords:** endometrial cancer, fiducial marker (FM), radiotherapy, adjuvant treatment, IGRT (image guided radiation therapy)

## Abstract

**Introduction:**

Intermediate-high and high-risk endometrial cancer often require adjuvant treatments such as radiotherapy (RT) or brachitherapy (BT) to reduce the risk of loco-regional relapse. Inter- and intra-fraction variability of internal pelvic anatomy are possibly the largest source of error affecting pelvic RT. The implantation of Fiducial Makers (FMs) in the vaginal cuff of patients receiving RT or BT could help patient daily setup, image guidance and intra-fraction detection of the radiation targets.

**Clinical case:**

We have evaluated the case of an 80-year-old woman treated with surgery for endometrioid adenocarcinoma G2 (stage pT1b Nx LVSI+) who underwent adjuvant pelvic IMRT after the implantation of vaginal cuff FMs.

**CT-simulation, Treatment Planning and IGRT strategy:**

Patient underwent planning CT scan 10 days after FMs implantation. RT consisted of 45Gy in 25 daily fractions to pelvic lymph nodes and surgical bed with simultaneous integrated boost up to 52.5Gy to the vaginal cuff and the upper two-thirds of the vagina. Cone beam Computed Tomography (CBCT) was acquired prior to every RT fraction for IGRT. Bladder and rectum were re-contoured on every CBCTs. Bladder and rectal volumes and median shifts were reported on a prospective database to quantify the impact of the pelvic organ variations.

**Results:**

The patient reported no discomfort during the FMs implantation, and no complications were seen. No evidence of FMs migration was reported. Bladder and rectal volumes planned contours were 245 and 55.3cc. Median bladder volumes for approved and “not acceptable” CBCTs were 222cc (range: 130-398) and 131cc (range: 65-326), respectively. Median rectal volumes for approved and “not acceptable” CBCTs were 75cc (range: 58-117) and 90cc (range: 54-189), respectively. The median values of the anterior-posterior, superior-inferior, lateral direction shifts were 3.4, 1.8 and 2.11 mm, respectively.

**Conclusion:**

In our clinical case, the implantation of FMs in the vaginal cuff of a patient who underwent pelvic adjuvant RT was well tolerated and reported no complications. The use of IGRT procedures based on FMs surrogating the vaginal vault may reduce inter-observer variability and pave the way for adaptive strategies or stereotactic treatments as external beam pelvic boost in gynecological field.

## Introduction

The majority of endometrial cancers (EC) are diagnosed at an early stage (80% in stage I), with 5-year survival rates of over 95% (1). However, 10% to 25% of women are initially diagnosed with more advanced disease (FIGO stages III and IV) and face considerable challenges, including multimodal adjuvant treatment with potentially serious morbidity and mortality (2).

When multimodal treatment is required, in addition to total hysterectomy with bilateral salpingo-oophorectomy and surgical lymph node staging (3), adjuvant external-beam radiotherapy (EBRT) is recommended, especially for substantial LVSI and/or for stage II, to reduce the risk of pelvic and para-aortic nodal relapse (3). Adjuvant brachytherapy (BT) alone can be considered for high-grade LVSI-negative and for stage II grade 1 endometrioid carcinomas (3). Moreover, when molecular classification is known, POLEmut and p53abn have specific recommendations: for patients with EC stages I–II, low risk based on pathogenic POLE mutation, omission of adjuvant treatment should be considered (3). The PORTEC-3 trial instead showed a statistically significant survival advantage for p53abn carcinomas with combined therapy (chemo + radiotherapy) for stages I–III (3, 4).

When recommended, radiotherapy should preferably commence within 6–8 weeks from surgery or be scheduled in relation to chemotherapy. In this context, the intensity-modulated radiotherapy/volumetric modulated arc therapy (IMRT/VMAT) technique results in a very precise dose that is targeted to the tumor while also reducing the exposure to surrounding organs. Indeed the combination of modulated treatments and daily image-based verifications (image-guided radiotherapy, IGRT) has helped to reduce gastrointestinal, genitourinary, and hematologic toxicities, permitting the use of smaller planning target volume (PTV) margins (5, 6).

Regarding BT, the target volume is individually determined and is usually the upper third of the vagina. When image-guided adaptive BT is applied, imaging of the applicator with CT scan or MRI evaluates whether the applicator is in close apposition to the vaginal mucosa and in proximity to OARs. This allows verification and calculation of cumulative doses, especially if vaginal BT is adopted as a boost after EBRT (6).

When delivering post-operative EBRT as adjuvant treatment for EC patients, vaginal cuff motion represents a critical issue for radiation oncologists. Indeed the position of the vaginal vault is reported to vary (up to 14.6 mm) due to a difference in daily intra/inter-fraction bladder and rectal fillings; using IGRT with implanted fiducial markers (FMs) as surrogates for the position of the vaginal–parametrial clinical target volume (CTV) (5, 7, 8) could avoid this issue.

This paper represents the first case in this specific subset of patients to assess the benefit of using FMs in the vaginal cuff of EC women receiving adjuvant radiotherapy.

## Clinical case

We present the clinical case of a patient treated with surgery for EC, who underwent adjuvant radiotherapy at our hospital (i.e., “Santa Maria della Misericordia” University Hospital, Udine, Italy) after the implantation of three gold fiducial markers in the vaginal cuff.

The patient was 80 years old at diagnosis, had no previous surgery, and had no comorbidity (except for cognitive decline). After an episode of vaginal bleeding in March 2022, she was diagnosed with EC. At 2 months later, she turned to our hospital and, according to the most recent guidelines, a laparoscopic total hysterectomy + bilateral salpingo-oophorectomy was performed; lymphadenectomy was not completed because of the patient’s age and there was no fluorescence evidence after indocyanine green injection in the cervix. Therefore, she was diagnosed with endometrioid adenocarcinoma G2 (stage pT1b Nx) with lymph-vascular invasion (LVSI+) and a molecular pattern of low-copy-number EC. After discussing the case at the multidisciplinary meeting, the patient was considered eligible for adjuvant RT with implanted FMs. Both preoperative imaging and tumor markers were negative for the patient. No intra- nor postoperative complications were observed, and the patient had an optimal radiotherapy tolerance. Currently, she is alive and disease-free. The patient’s characteristics are summarized in **Table 1**.

**Table 1:** Patient’s features.

Patient’s features	
Age	80
Previous abdominal surgery	None
Comorbidities	Cognitive decline
Imaging and markers	Negative
Symptoms	Vaginal bleeding
Hysteroscopy	Yes
Type of surgery	Total hysterectomy + bilateral salpingo-oophorectomy LPS
Diagnosis	Endometrioid adenocarcinoma
Grading	G2
Staging	pT1b Nx LVSI+
Molecular pattern	Low-copy-number endometrial cancer

In July (about 3 weeks before treatment), three gold FMs (0.40 mm × 10 mm) (Gold Anchor™ Naslund Medical AB, Huddinge, Sweden) were implanted in the vaginal cuff of the patient.

From August 1 to September 12, the patient underwent adjuvant IMRT consisting of 45 Gy in 25 daily fractions (1.8 Gy per fraction) on the surgical bed and pelvic lymphatic drainage with a simultaneous integrated boost (SIB) of up to 52.5 Gy (2.1 Gy per fraction) on the vaginal cuff.

## Procedural method—insertion of markers

**Figure 1:**
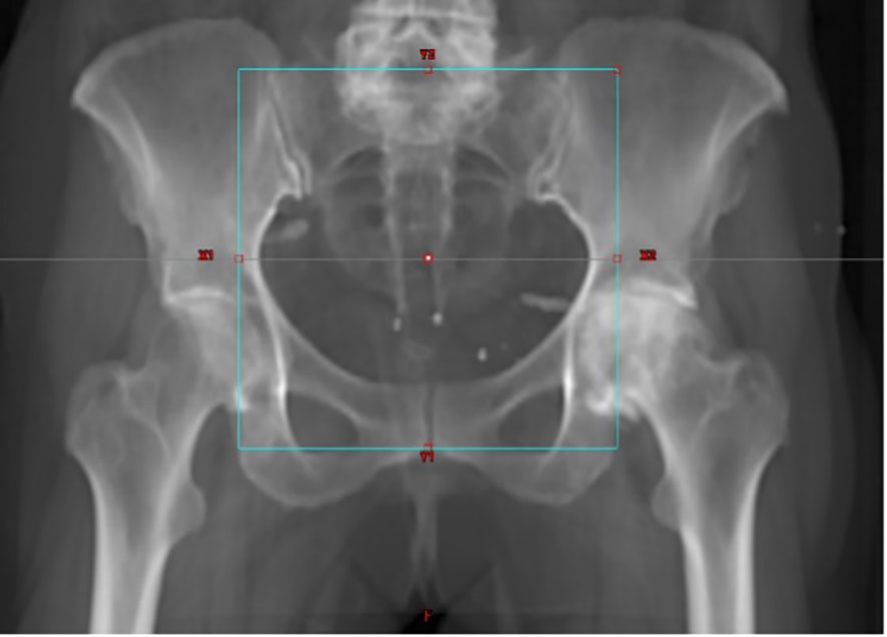
Digitally reconstructed radiograph in antero-posterior direction.

After providing informed written consent, the FMs’ implantation procedure was performed by a gynecologist, in an outpatient setting, with the patient in gynecological position. Before the procedure, a local anesthesia was used with the application of lidocaine spray on the vaginal cuff, and a transvaginal ultrasound was performed for proper evaluation of the target area. Fiducial markers were inserted in the vaginal cuff with the following steps: the first marker was placed on the bevel of a 20 gauge 20-cm injection needle, the needle was inserted in the target area, and finally, the marker was released at this level. The same procedure was repeated for the other two fiducials. The setup of the three fiducials allowed triangulation and the measurement of position in different planes and provided a surrogate for the position of the vaginal cuff (**Figure 1**).

At the end of the procedure, the patient did not report pain or discomfort. The patient underwent a low-dose CT scan at a CT simulator to verify the correct positioning of FMs. No antibiotic prophylaxis was required nor cessation of anticoagulant/antiplatelet medications.

## CT simulation and treatment planning

**Figure 2:**
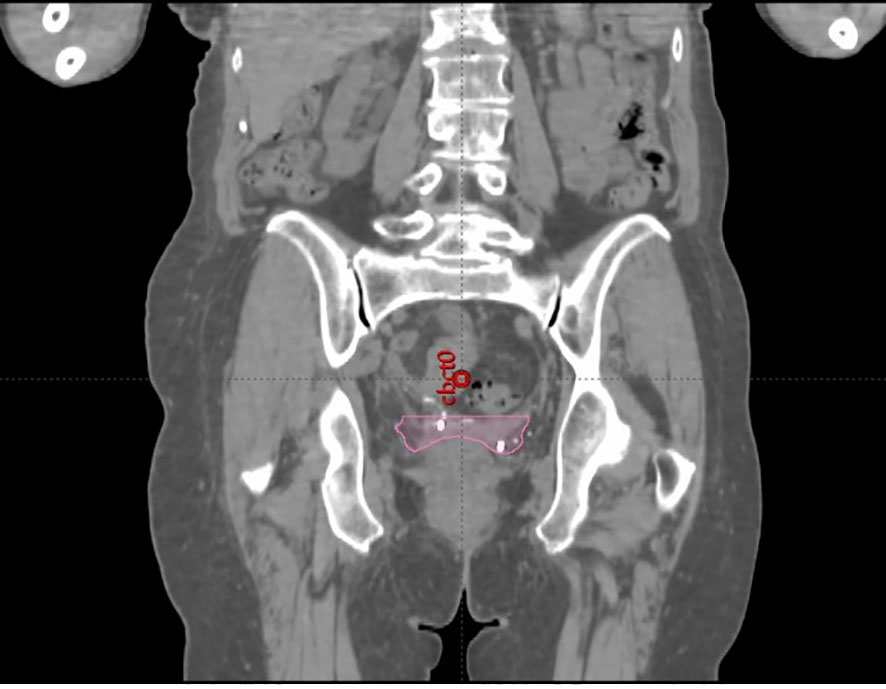
Planning CT image with the vaginal CTV with FMs.

The patient underwent a planning CT scan using a Philips Brilliance Big Bore CT simulator (Phillips Medical Systems, The Netherlands), 10 days after the implantation of the fiducials. A 120-kV and 2-mm-slice-thickness scan protocol was used. No intravenous contrast was administered. The CT images were acquired with the patient in the supine position, headfirst to gantry and immobilized with Combifix™ frame (Civco Inc.^®^). The lower limit of the scanned area was set to 2 cm below the lower limit of the lesser trochanter; the upper limit was the L2–L3 interspace. The patient underwent a bladder filling protocol and empty rectum/bowel prior to the planning CT to ensure a consistent bladder volume for the scans and treatment. She was asked to drink 500 cc of water 30 min before planning CT and before each treatment session. The pelvic nodal CTV (obturator lymph nodes, external iliac nodes, internal iliac nodes, and presacral nodes) was defined with a 6- to 7-mm uniform margin surrounding arteries and veins and excluding bones and muscles. Surgical bed/vaginal CTV was defined as the upper two thirds of the vagina, the parametrial–paravaginal tissues. A 5-mm isotropic expansion to CTV was used to obtain a pelvis-planning target volume receiving a total prescribed dose of 45 Gy in 25 fractions (PTV45). A second volume including the vaginal cuff and the upper two-thirds of the vagina was defined for the integrated boost (**Figure 2**). The corresponding PTV52.5 (SIB-PTV) was generated using an isotropic margin of 5 mm.

Volumetric arc therapy (VMAT) treatment plan was designed for the patient, using the Eclipse™ Treatment Planning System (Version 15.5; Varian Siemens), with a 6-MV photon beam (TrueBeam STx; Varian Siemens). The plan consisted of two full-arcs (clockwise and counterclockwise) with complementary 30° collimator angles.

The treatment plan aimed to cover ≥95% of the volume of PTVs with the prescribed dose and to restrict a maximum dose of <110% of the prescribed dose.

The target volumes and OARs, including the bladder, the bowel defined as intestinal cavity, rectum, and femoral heads were delineated according to the Radiation Therapy Oncology Group (RTOG) consensus panel atlas (6). No adaptive radiotherapy was planned for the treatment.

## IGRT strategy

RT was delivered as one fraction/day for 5 days per week. The identical bowel and bladder procedure from the CT simulation as well as the pelvic instructions were repeated for the daily treatments. With the use of an in-room laser coordinate system, the patient was immobilized and positioned utilizing the four-point setup tattoos.

Prior to daily treatment delivery, a full pelvic cone beam computed tomography (CBCT), with the same slice thickness of the CT simulation (2 mm), was performed. The use of the CBCT had a double purpose: to check the volume of the bladder and rectum and to verify the patient setup. Planning CT and CBCT images were automatically rigidly matched based on bone anatomy, including the femoral heads, lower lumbar vertebrae and ischial tuberosity, sacrum, and pubic symphysis. A first 6DoF (degree of freedom) registration was done to check the patient setup; if the rotations (pitch/roll and yaw) deviations are greater than 3° and 1°, respectively, the patient was repositioned; otherwise, a 3DoF match (limiting on the translation axes) was performed.

After a visual inspection of the automatic registration by an expert radiation oncologist, additional manual corrections were performed to improve the match in the target area, focusing on the fiducial marker implanted in the vaginal vault.

If the rectum and bladder filling were not appropriate, the patient was asked to empty her bowels, and the CBCT was repeated. If the rectum and bladder filling were adequate, the registration triggered the treatment when the pelvic bones matched within 5 mm, and the couch shifts were applied remotely for all axes whenever a correction on any single axis was ≥1 mm. Otherwise, the patient was repositioned. After the on-line correction, a verification CBCT was acquired. The results of the registration procedure (couch shifts) were recorded.

Using the On Board Imaging system and the advanced IGRT motion package available on the Varian Truebeam v2.7 and later Siemens Varian, kV images were acquired during arc delivery for intrafraction motion management. The software searches for marker locations on kV images acquired with OBI and gives quantitative feedback on how close the markers are to the expected location. As a feasibility study, we observed the *in vivo* FM imaging, but no action was based on them.

At the end of delivery, another CBCT acquisition was made for study purposes to check the filling state of the rectum and bladder.

For the longest treatment sessions, another CBCT was performed at the end of the arc delivery to study the filling state of the rectum and bladder.

To quantify the actual impact of the variations of the bladder and rectum volumes on the accuracy of the registration (and then on the dose distribution), the bladder and rectum were re-contoured on every CBCT by the same expert radiation oncologist, and their volumes were reported in a prospective database.

## Results

From August 1 until September 12, 2022, the patient underwent 25 daily EBRT fractions. During this period of time, no evident migration of the markers was observed.

A total of 63 CBCTs and their time were registered. After acquisition, 11 (44%) CBCTs were deemed not to be acceptable at the first attempt for delivering the treatment due to unsatisfactory bladder–rectum preparation. In particular, among those, two CBCTs showed a full rectum (feces or gas), six showed insufficient bladder filling, and three showed both a full rectum and an empty bladder.

The median value (as absolute value) of the shift in the anterior–posterior (AP) direction was 3.4 mm (range: -7.0 to 1.7 mm), while in the superior–inferior shift (SI) and in the lateral direction, the median value (absolute value) was 1.8 mm (range: -0.47 to 3.4 mm) and 2.11 mm (range: -0.89 to 4.6 mm), respectively.

The bladder and rectal volumes contoured on planning CT were 245 and 55.3 cc, respectively. The median bladder volume for approved CBCTs was 222 cc (range: 130–398 cc). The median bladder volume for “not acceptable CBCT” was 131 cc (range: 65–326 cc). The median rectal volume for approved CBCTs was 75 cc (range: 58–117 cc). The median rectal volume for “not acceptable CBCT” was 90 cc (range: 54–150 cc).

## Discussion

It is well known that intermediate-high- and high-risk EC often require adjuvant treatments such as RT or BT to reduce the risk of local relapse (3–8). With the routine implementation of advanced RT techniques, image guidance is increasingly important. The current standard is using IMRT to reduce toxicity and to ensure high dose conformity to target volumes. However, multiple sources of uncertainty still exist, compensated mostly by treatment volume margins. Day-to-day and intrafraction variability of internal pelvic anatomy relative to the planning imaging are possibly the largest source of error. The implantation of FM in the vaginal cuff of patients receiving EBRT or BT could help the daily patient setup, image guidance, and intra-fraction detection of the radiation targets. Within the field of radiotherapy for gynecological cancer, there are some studies in literature assessing the role of fiducial markers on helping to outline the target tissues (7–11). FM implantation has been used by Jhingran et al. to estimate variations in vaginal apex position according to changes in rectal and bladder filling reporting significant shifts in all directions, especially in the anterior–posterior direction (9). Rash et al. analyzed 145 daily CBCTs from five patients with gold FMs implanted in a vaginal vault, reporting in one patient FM displacement outside PTV in 16% of the treatment (10). Two-year clinical outcomes of vaginal FMs–IGRT treatment on 26 patients were published by Monroe et al., showing significant transnational shifts from a clinical setup which required a median correction of 9.1 mm. They reported a low incidence of acute GI/genitourinary (GU) toxicity with no relapse at 2 years (11). FMs may be useful for adaptive radiotherapy strategy, as investigated by Buijs et al. (8).

In this case report, we describe our experience in the implantation procedure of three gold FMs in the vaginal cuff of a patient surgically treated for EC and her subsequent radiotherapy. We observed that the markers’ implantation did not require any hospitalization and that it has been a quick and safe procedure. We have not experienced markers’ migration, and the patient did not report any discomfort. In our preliminary experience, particular attention was paid to the role of the FMs in the IGRT procedure. We adopted an IGRT strategy in the gynecological setting, mimicking the prostate–RT paradigm in which the FMs are widely used (12). Also, in our center, this kind of IGRT represents the routine practice in 90% of the prostate patients since 2010.

Analyzing the collected data (IGRT data and OAR volumes), we observed how bladder and rectum fillings have a significant impact on the position of the vaginal cuff represented by the FMs. Once the correct rectum and bladder preparation was obtained, the match between planning CT and CBCT resulted to be improved, in particular referring to FMs having a residual target error inferior to 2 mm.

## Conclusion

This manuscript aimed to assess the feasibility and the safeness of the implantation of gold fiducial markers in the vaginal cuff of a patient who had to undergo pelvic adjuvant radiation therapy. In our clinical case, the procedure reported no complications (no pain, no bleeding, no inflammation, and no infections) and was well tolerated by the patient. Accuracy in treatment preparation is of utmost importance for the quality and safety of radiation treatment. Beyond this, the use of standardized volumetric IGRT procedures based on implanted fiducial markers surrogating the vaginal vault may help to reduce inter-observer variability and paves the way for adaptive strategies or stereotactic treatment for pelvic boost external irradiation or cervix or recurrent carcinoma in gynecological cancer patients. A confirmatory perspective study is ongoing in our center to support these results.

## Data availability statement

The original contributions presented in the study are included in the article/supplementary material. Further inquiries can be directed to the corresponding author.

## Ethics statement

The studies involving humans were approved by the Institutional Review Board (IRB-DAME)–Udine–UNIUD. The studies were conducted in accordance with the local legislation and institutional requirements. The participants provided their written informed consent to participate in this study. Written informed consent was obtained from the individual(s) for the publication of any potentially identifiable images or data included in this article.

## Author contributions

SR and FTi: conceptualization and original draft preparation. MA, EM, FTo, and GP: revision of the manuscript. AP, GVu, CP, and EB: data curation and methodology. GVi: conceptualization and supervision.
